# Successful Zanubrutinib Monotherapy in a Rare CNS Presentation of Relapsed CLL

**DOI:** 10.1155/crh/1150546

**Published:** 2026-01-06

**Authors:** Vishaal Kunta, Mohammad Ammad-Ud-Din, Melanie Mediavilla-Varela, Javier Pinilla-Ibarz

**Affiliations:** ^1^ Department of Immunology, H. Lee Moffitt Cancer Center and Research Institute, 12902 USF Magnolia Drive, Tampa, 33612, Florida, USA, moffitt.org; ^2^ Department of Malignant Hematology, H. Lee Moffitt Cancer Center and Research Institute, 12902 USF Magnolia Drive, Tampa, 33612, Florida, USA, moffitt.org

## Abstract

**Background:**

Central nervous system (CNS) involvement is an infrequent complication of chronic lymphocytic leukemia (CLL), occurring in less than 1% of cases. We report a case of a 63‐year‐old male with a history of CLL previously treated with ibrutinib but discontinued early due to intolerance. As a result, the patient was then treated with obinutuzumab plus venetoclax, achieving undetectable minimal residual disease (MRD) but relapsed after 2 years with CNS involvement.

**Case Presentation:**

The patient initially presented to the emergency department with confusion and altered mental status. Magnetic resonance imaging (MRI) of the brain revealed abnormal subcortical hyperintensities and leptomeningeal enhancement concerning leukemic infiltration. Lumbar puncture confirmed malignant CD5+ CLL cells in the cerebrospinal fluid (CSF), and a bone marrow biopsy revealed 50%–60% CLL involvement. Zanubrutinib 320 mg daily was initiated. The patient exhibited marked cognitive improvement within full resolution after four weeks of therapy. Follow‐up MRI after 8 weeks showed full resolution of CNS, lesions with repeat LP demonstrating CSF cleared of CLL cells. He remains in complete remission with continued daily zanubrutinib 6 months follow‐up; no significant adverse effects were observed.

**Conclusion:**

This case highlights the rare occurrence of CNS involvement in CLL and is the first to demonstrate successful CNS disease eradication with zanubrutinib monotherapy.

## 1. Introduction

Chronic lymphocytic leukemia (CLL) is the most common adult leukemia in Western countries, typically affecting older adults (median age around 70 years) [1, 2]. It is characterized by clonal accumulation of mature CD5+ B‐lymphocytes in the peripheral blood, bone marrow, and lymphoid tissues [1]. The disease course is usually indolent, with many patients managed initially by observation. Standard first‐line treatments, when indicated by symptomatic disease or cytopenias, have evolved from chemoimmunotherapy (e.g., fludarabine, cyclophosphamide, and rituximab) to targeted agents, including BTK inhibitors (ibrutinib, acalabrutinib, and zanubrutinib) and the BCL2 inhibitor venetoclax (often combined with anti‐CD20 antibodies) [1, 3]. These novel agents have significantly improved CLL outcomes.

Despite improved sensitivity of CLL diagnosis, CLL rarely involves extranodal sanctuary sites. In particular, CNS involvement by CLL is an infrequent event [2]. While historical autopsy studies indicated occult CNS infiltration in up to 70% of CLL cases, clinically symptomatic “leukemic meningitis” or brain parenchymal infiltration is reported in less than 1% of patients [1, 2]. There are no well‐defined risk factors for this condition since CNS involvement can occur at any time in the disease course (median around 2.6 years from CLL diagnosis) and does not correlate with high‐risk cytogenetics or systemic CLL burden [1]. Patients can present with a variety of symptoms such as headaches, confusion, cranial neuropathies, or other neurological deficits, often leading to a diagnostic workup including MRI and CSF analysis [2]. Given its rarity, there is no consensus treatment for CLL with CNS involvement [2]. Reported approaches have included intrathecal chemotherapy (methotrexate or cytarabine) and/or cranial irradiation, often extrapolated from other CNS lymphomas [4]. More recently, small‐molecule therapeutics that cross the blood‐brain barrier (such as ibrutinib, pomalidomide, and venetoclax) have shown activity in individual cases [1, 2]. However, outcomes have been variable, and CLL with CNS involvement historically portended a poor prognosis with a median survival of around 9–10 months [1]. Early recognition and intervention are thought to improve outcomes, and achieving clearance of CLL from the CSF has been associated with prolonged survival in retrospective series [1, 4].

Zanubrutinib is a highly potent, selective, irreversible BTK inhibitor that inhibits B‐cell receptor signaling, thereby inducing apoptosis or anergy in CLL cells [5, 6]. Compared to the first‐generation BTK inhibitor ibrutinib, zanubrutinib has greater BTK specificity with fewer off‐target effects (e.g., less inhibition of EGFR and TEC kinases) and a longer half‐life, allowing sustained BTK inhibition [3, 7, 8]. These pharmacologic features translate into improved tolerability, as indicated by lower rates of atrial fibrillation, bleeding, and deep target inhibition [8–12]. Notably, emerging evidence indicates that zanubrutinib achieves clinically meaningful CNS penetration [13]. BTK inhibitors, as a class, can cross the blood‐brain barrier. Ibrutinib has documented efficacy in CNS lymphomas and CLL with CNS involvement. However, zanubrutinib has shown high blood‐brain barrier permeability, exceeding that of earlier agents [14]. Currently, zanubrutinib is approved for CLL/SLL and other B‐cell malignancies, but its use specifically for CNS involvement of CLL is novel. To our knowledge, no prior published case reports of zanubrutinib monotherapy successfully treating CLL with CNS involvement exist. Here, we present the first case, highlighting the rarity of CLL CNS involvement and the efficacy of single‐agent zanubrutinib as a targeted CNS‐active therapy.

## 2. Case Presentation

A 63‐year‐old male was initially diagnosed with CLL when he was presented with lymphocytosis. At diagnosis, fluorescence in situ hybridization (FISH) revealed del(11q) and del(13q) chromosomal abnormalities, and the patient had an unmutated IGHV gene status. He initially had Rai Stage II disease. Given the 11q deletion and symptomatic nodes, first‐line therapy with the BTK inhibitor ibrutinib at 420 mg was started, but a week after, he was admitted for an acute onset of atrial fibrillation that required rate control and anticoagulation. At the time, the patient did not want to continue on reduced doses of ibrutinib and was switched to therapy with obinutuzumab plus venetoclax (O + V) for one year. This combination led to a deep response through which the patient attained complete remission with undetectable minimal residual disease (uMRD) by next‐generation sequencing (NGS) after 12 months. The patient then entered an off‐treatment surveillance phase.

Two years later, the patient presented to the clinic with a 3‐week history of cognitive decline, memory impairment, and personality changes. No headaches or seizures were reported, but the family did note progressive confusion. On exam, he was oriented but exhibited slowed cognition and mild right‐sided weakness. Given their CLL history, CNS involvement was considered. Brain MRI demonstrated multiple patchy FLAIR hyperintensities in the bilateral frontal and parietal lobes with diffuse leptomeningeal contrast enhancement, findings concerning leukemic involvement of the CNS (Figure 1(a)). A lumbar puncture was performed, and CSF analysis showed 28 leukocytes/μL (80% lymphocytes) with elevated protein (70 mg/dL). Flow cytometry of CSF revealed a clonal B‐cell population (CD19^+^, CD5^+^, CD23^+^, and kappa light chain–restricted) consistent with CLL involvement. Cytology also showed small mature lymphocytes. No high‐grade large cells were seen, arguing against Richter’s transformation. Concurrent systemic evaluation revealed CLL involvement in the bone marrow (approximately 50%–60% lymphocytes infiltration on biopsy). Peripheral blood lymphocyte count had risen to 45 × 10^9^/L from its lowest level of < 1 × 10^9^/L a year prior. Notably, there was no significant lymph node enlargement on imaging and no other extranodal disease. The diagnosis was established as a CNS involvement of CLL (leptomeningeal CLL) concurrent with bone marrow relapse.

Figure 1(a) Axial section of MRI brain with contrast (TW‐weighted) showing extensive leukemic involvement with a large confluent area of enhancing restricted diffusion and increased FLAIR and T2‐weighted signal change extensively involving the supratentorial brain and posterior fossa. (b) Complete resolution of the previously noted extensive T2‐weighted enhancement in the supratentorial brain and posterior fossa compatible with response to treatment.

**Figure:**
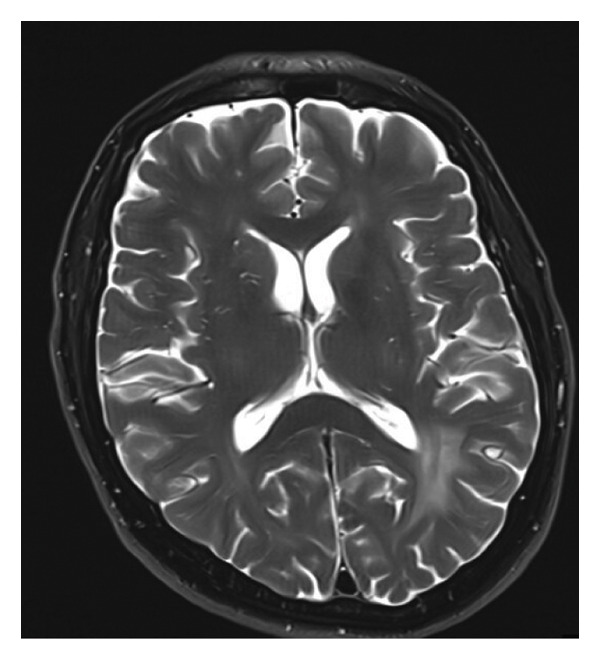
(a)

**Figure:**
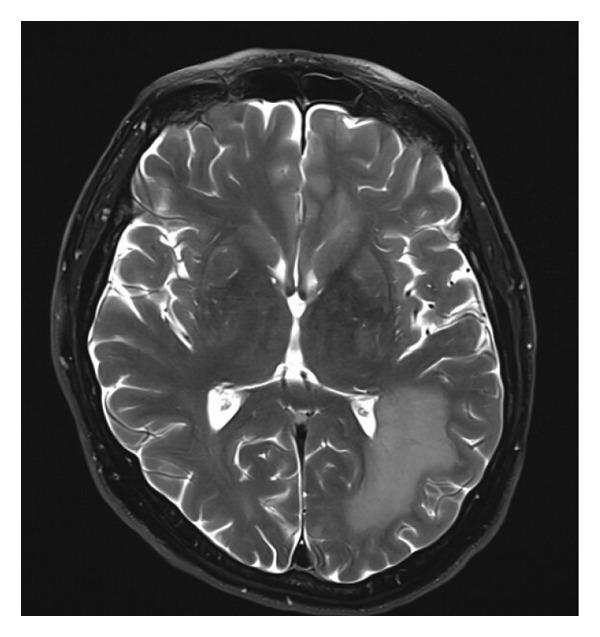
(b)

After multidisciplinary discussion, zanubrutinib 320 mg daily was determined to be the sole treatment. This decision was influenced by the patient’s prior therapy history and overall condition. He had BTK intolerance and relapse after a BCL2 inhibitor‐based therapy. Although intensive approaches like high‐dose methotrexate were considered, based on the low proliferative rate of CLL cells and possible associated toxicity, it was not a good option. No intrathecal chemotherapy was given.

The patient’s neurologic status began improving within two weeks of starting zanubrutinib. By 6 weeks of treatment, his family noted a return to near‐normal cognitive function. He was more alert and oriented and could carry out daily tasks without confusion. After 2 months of therapy, follow‐up brain MRI showed a complete resolution of the prior T2/FLAIR lesions and leptomeningeal enhancement, with no new abnormalities, indicating radiographic remission of CNS CLL (Figure 1(b)). A repeat lumbar puncture at that time was acellular, and CSF flow cytometry was negative for clonal B‐cells, indicating clearance of the disease. A second MRI was given and showed continued response. At the 11 months follow‐up, the patient continues to be in complete remission and is tolerating zanubrutinib with no adverse effects.

## 3. Discussion

CNS involvement in CLL is an uncommon and challenging clinical scenario. This case underscores two essential and novel aspects: the rarity of CLL with CNS involvement and the successful use of zanubrutinib monotherapy to achieve CNS disease clearance. Only a tiny fraction of CLL patients (< 1%) develop symptomatic CNS involvement by their leukemia [2, 3]. A variety of presentations have been documented, ranging from leptomeningeal disease causing encephalopathy (as in our patient) to isolated cranial nerve palsies or even parenchymal brain masses [2, 4]. Because these symptoms often arise in older patients who frequently have other comorbidities, diagnosis of CLL CNS involvement can be delayed or missed [1]. Our patient’s initial symptom of confusion, for example, had a broad differential diagnosis. The diagnosis is typically confirmed by CSF analysis showing CLL cells (by flow cytometry or cytology) and by neuroimaging findings [2]. In our case, MRI and CSF studies were crucial in confirming the diagnosis of leptomeningeal CLL. It is worth noting that the patient’s prior history of lymphoma raised concern for a possible Richter’s transformation in the CNS; however, the CSF lacked large atypical cells, and immunophenotyping matched his CLL clone, consistent with a direct CLL relapse. This aligns with literature indicating that while Richter’s transformation can involve the CNS (typically as diffuse large B‐cell lymphoma), most CLL CNS cases are due to infiltration by the original CLL clone [4].

Optimal therapy for CLL with CNS involvement is not established, given the rarity of the condition and the absence of prospective trials. Historically, approaches have mirrored treatments for other leukemic or lymphomatous meningitis. Intrathecal chemotherapy (such as methotrexate or cytarabine) is commonly used in reported cases, often alongside systemic therapy for overall disease control [2, 4]. Whole‐brain or meningeal radiation has also been employed in cases of localized CNS disease or persistent meningeal involvement [4]. These traditional approaches can be effective but carry risks. Intrathecal chemotherapy may cause neurotoxicity, and radiation can lead to cognitive decline, especially in older patients. In recent years, targeted agents have shown promise in this arena. Bruton tyrosine kinase inhibitors (BTKi) have emerged as attractive options because of their ability to penetrate the CNS and their systematic action against CLL. Ibrutinib, a first‐generation BTKi, has several reports documenting its use in CLL with CNS involvement, which can sometimes lead to disease control without the need for intrathecal therapy [2, 15]. Likewise, immunomodulatory agents such as lenalidomide/pomalidomide, which cross the blood‐brain barrier, have been combined with chemotherapy to treat CLL or Richter’s syndrome in the CNS [2]. The BCL2 inhibitor venetoclax, despite high protein‐binding, has also shown activity in CLL CNS disease in at least one case report [1]. However, these approaches have been described in only a handful of cases. A 2017 retrospective study of 30 patients with CNS CLL noted varied treatments and responses, highlighting the need for individualized therapy [4, 16].

Our decision to use zanubrutinib monotherapy in this patient was driven by clinical practicality and emerging evidence of BTK inhibitor efficacy in CNS malignancies [13, 17]. Importantly for our case, zanubrutinib’s pharmacokinetic profile (high oral bioavailability, sustained plasma levels with 160 mg BID dosing) and greater BTK selectivity allow for effective dosing without dose‐limiting toxicities [3, 7]. This likely enabled adequate drug penetration into the CSF. Although CNS drug level data for zanubrutinib in humans are limited, preclinical models and clinical experience in diseases like Waldenström macroglobulinemia suggest meaningful CNS activity [7]. In a reported case of Bing–Neel syndrome (CNS involvement by Waldenström’s), single‐agent zanubrutinib led to neurological improvement and MRI‐confirmed resolution of spinal cord lesions [7]. Notably, we avoided intrathecal chemotherapy and achieved clearance of malignant cells from the CSF. This highlights the penetrative ability of zanubrutinib and suggests that this option can sometimes suffice for CNS CLL, simplifying treatment and reducing risk to the patient.

It is instructive to compare our case with prior reports. In many earlier cases, combination approaches were used, such as high‐dose methotrexate plus ibrutinib or intrathecal therapy combined with systemic chemo [2]. Our patient’s excellent response to monotherapy highlights the potency of BTK inhibition. The absence of neurologic side effects or systemic toxicities in our patient also speaks to zanubrutinib’s favorable safety. We did monitor closely for known class effects such as minor bleeding or cytopenias, but none occurred.

One case reported using zanubrutinib as a monotherapy for CNS involvement of CLL, but the patient only showed a partial response with limited clinical recovery, despite radiologic improvement [1]. There could be a couple reasons for the reduced success in treatment. The patient experienced a gastrointestinal hemorrhage, necessitating early discontinuation of therapy. Additionally, clinical trajectory was complicated by the patient’s concurrent conditions like HHV‐6 encephalitis and hyponatremia, which likely contributed to the patient’s progressive encephalopathy. While imaging showed modest improvement, the patient’s health continued to deteriorate, leading the family to pursue comfort‐focused care. It remains difficult to delineate the extent to which the patient’s neurologic decline was attributable to leukemic infiltration versus viral encephalitis.

Our case also demonstrates that prior exposure to a different BTK inhibitor (ibrutinib) without evidence of disease progression does not preclude a successful response to zanubrutinib. Cross‐resistance between covalent BTK inhibitors can occur if the CLL acquires a BTK C481S mutation or PLCG2 mutation [3]. In our patient, who had discontinued ibrutinib for intolerance rather than disease progression, no such resistant clone was likely selected, allowing zanubrutinib to be effective. Even in patients who develop BTK mutations, noncovalent BTK inhibitors like pirtobrutinib have shown efficacy and could be considered if zanubrutinib fails [3]. The field of CLL is rapidly evolving, and targeted therapies are increasingly being applied to traditionally hard‐to‐treat scenarios like CNS involvement.

Our experience suggests that in CLL patients with CNS involvement, a trial of a CNS‐penetrant agent such as zanubrutinib is a reasonable initial strategy. It offers a less invasive approach compared to intrathecal chemotherapy or radiation. Close monitoring is vital for toxicities like bleeding. If an inadequate response is observed, escalation of therapy can be considered in patients with robust performance status, for instance, by adding intrathecal methotrexate or considering novel agents or clinical trials. Given the complex nature of CNS leukemia management, multidisciplinary care involving hematology, neurology, and oncology specialists is recommended.

## 4. Conclusion

Our case highlights the potential efficacy of zanubrutinib monotherapy in treating relapsed CLL with CNS involvement. While the favorable clinical response suggests zanubrutinib may be active in this setting, larger studies utilizing multicenter databases are needed to better define its long‐term effectiveness, safety, and impact on survival. Such data will be essential to guide treatment strategies for this uncommon but serious manifestation of CLL.

## Consent

No patient identifiers were used, and the patient was sufficiently anonymized according to ICMJE guidelines and aligns with CARE checklist criteria. No written consent has been obtained from the patients as there is no patient identifiable data included in this case report.

## Conflicts of Interest

Javier Pinilla‐Ibarz reports receiving consulting fees and honoraria from Beigene, Abbvie, AstraZeneca, and Lilly. The remaining authors declare no conflicts of interest.

## Author Contributions

Javier Pinilla‐Ibarz was directly involved with the patient’s care. Javier Pinilla‐Ibarz, Mohammad Ammad‐Ud‐Din, and Vishaal Kunta wrote the original draft and reviewed and edited the manuscript. Melanie Mediavilla‐Varela provided clinical information and edited the manuscript. Javier Pinilla‐Ibarz supervised the work. Vishaal Kunta and Mohammad Ammad‐Ud‐Din contributed equally to this study and share co‐first authorship.

## Funding

This research received no specific grant from any funding agency in the public, commercial, or not‐for‐profit sectors.
